# Prison sentencing increases the risk of unemployment among illegal heroin users in Taiwan

**DOI:** 10.1186/s13011-020-00320-3

**Published:** 2020-10-12

**Authors:** Charles Tzu-Chi Lee, Chiu-Mieh Huang, Li-Chun Chang, Shih-Wen Wang, Hsiao-Pei Hsu, Jung-Yu Liao, Jong-Long Guo

**Affiliations:** 1 Department of Health Promotion and Health Education, National Taiwan Normal University, 162, Sec. 1, He-ping East Road, Taipei, Taiwan 10610; 2 Institute of Clinical Nursing, School of Nursing, National Yang-Ming University, 155, Sec. 2, Li-Nong Street, Taipei, Taiwan 11221; 3 Department of Nursing, Chang Gung University of Science and Technology, 261, Wen-Hua 1st Rd, Tao-Yuan, Taiwan 33303; 4 Institute of Population Health Sciences, National Health Research Institutes (NHRI), 35 Keyan Road, Zhunan, Miaoli County 35053 Taiwan

**Keywords:** Deferred prosecution, Observation and rehabilitation, compulsory rehabilitation, Prison sentence, Employment rate, Heroin users

## Abstract

**Background:**

Previous studies have rarely explored the effect of type of sentencing on employment status among illegal heroin users, therefore, we aims to examine the association of the sentencing types and employment outcomes among illegal heroin users in Taiwan.

**Methods:**

Participants with illegal heroin use were identified through the national prison register system and deferred prosecution system: 2406 with deferred prosecutions, 4741 with observation and rehabilitation, 15 compulsory rehabilitation and 1958 sentenced to prison in calendar 2011. Logistic regression models were built to estimate the effect of sentencing type on unemployment status at 2 years after release. Stratification analysis was conducted to determine the effect of sentencing type based on the offender’s employment status before sentencing.

**Results:**

Illegal heroin users receiving a prison sentence were more than twice as likely to be unemployed 2 years later than those receiving deferred prosecution. The unemployment rate was also higher for those with observation and rehabilitation and compulsory rehabilitation than deferred prosecution in the 2 years following sentencing. Males, older users, without a job before sentencing, divorced or widowed and higher prior drug use criminal records were also higher risk of unemployment. Subgroup analysis by prior employment status revealed that being sentenced to prison, observation and rehabilitation and compulsory rehabilitation affected the subsequent employment status only for those heroin users with a job before sentencing. The strength of associations showed dose-dependent relationship between different sentencing types (sentenced to prison> compulsory rehabilitation> observation and rehabilitation) and employment outcomes.

**Conclusions:**

Illegal heroin users who receive a prison sentence have a much higher risk of unemployment than those who receive deferred prosecution after controlling potential confounders, especially those who had a job before sentencing. The implication is the stronger freedom of punishment, the higher risk of unemployment outcomes. Our study support that illegal heroin user is legally regarded as a patient before being regarded as a criminal, so giving priority to quit addition rather than imprisonment.

## Background

Illegal drug use and addiction represent a global crisis, creating seriously harm to the addict’s physical and mental health [1], social function and employment [2], even endangering the social order by increasing the rates of both crime and violence [3, 4]. For these reasons, numerous countries are committed to implementing programs to prevent, control and treat the use of illegal drugs. In 2018, according to the 2020 World Drug Report, 269 million people aged 15–64 years used at least one illegal drug in the previous year, an estimated 5.4% of the population; the most used illegal drugs are cannabis, opioids and opiates [5]. The prevalence of illegal drug use in Taiwan was approximate 1.2%, according to Taiwan’s first national household survey; the top five most used illegal drugs were methamphetamine, ecstasy, ketamine marijuana and heroin [6]. Heroin in particular is one of the world’s most widely used illegal drug [7]. According to the Taiwan Surveillance System of Drug use and Addiction Treatment, heroin is the one of the most commonly used drug in Taiwan; among drugs monitored, heroin accounted for 80.9% of illegal drug users who received treatment in 2002, a rate which rose to 93.8% in 2007, then declined to 83.3% in 2011 [8]. These percentages were more than 80% of illegal drug user received treatment.

Use of heroin and other substances burdens both the individual user and society as a whole, to a greater extent than other mental disorders [9]. Physical and mental disorders resulting from illegal heroin use may increase the medical cost, but this represents only be a small portion of the total cost of heroin dependency. For example, one study found that the medical cost of heroin dependency (23% of the total cost) is much smaller than the productivity loss (53% of the total cost) [10]. To this should be added the cost to the individual users (including financial expenses and health risks) as well as the social costs of crime to support the drug behavior [11].

Previous studies have shown that illegal drug use treatment reduced substance use and criminal behavior and improved the social functioning, employment, and physical and emotional health of the former user [12, 13]. The drug treatment alternative to prison program in Taiwan started as a deferred sentencing model, assisting illegal drug user into treatment. A 5-year study of the program indicated that the re-arrest rates and reconviction rates of those with deferred sentencing were significantly lower than those of offenders who received regular processing into the criminal justice system [14]. Taiwan also initiated a deferred prosecution and methadone maintenance treatment (MMT) pilot project to address the human immunodeficiency virus (HIV) epidemic. A shift in the official view of illegal drug users from “criminal” to “patient” in Taiwan suggested the corresponded strategic approach from “punishment” to “treatment.” Before 1998, all illegal drug users in Taiwan were sentenced as “criminals” to criminal penalties. After 1998, the Against Narcotics Act classified illegal drug users as “patients,” eligible for treatment and other non-sentencing interventions. Consequently, there was a deferred prosecution policy which did not immediately imprison the illegal drug users, but prefer to provide treatment [15]. Thus, illegal drug users in Taiwan may receive one of four types of sentence: (1) deferred prosecution and complete MMT, (2) observation and rehabilitation, (3) compulsory rehabilitation and (4) prison sentence. This sentencing aim to help illegal heroin users return to society, resume employment and live a normal life. It has replaced imprisonment with medical treatment, offers heroin offenders MMT, improves social interactions, and helps former users transition back into society.

For the illegal heroin user who was first seized by the police, the illegal drug user indicated to the prosecutor that he / she had the intention to quit addiction. If the prosecutor thought that he / she was suitable to stay in the community to quit addiction he / she was commanded to receive deferred prosecution and complete **MMT**. If the illegal drug user does not express his / her intention to quit addiction, the prosecutor will command the illegal heroin user to receive observation and rehabilitation with a period of less than 2 months. If there is no tendency to indicate the user continue using heroin, he / she will not be prosecuted at the end of the observation and rehabilitation. If there is a tendency to continue to use heroin after professional evaluation, he / she will receive a compulsory rehabilitation which is a treatment with a period more than 6 months until there is no need to continue compulsory rehabilitation. However, it may not exceed 1 year. Recidivists within 5 years may be prosecuted or applied for summary judgment, but prosecutors can still give deferred prosecutions and complete MMT if illegal heroin user indicates to the prosecutor that he or she has an intention to quit addition. Illegal heroin user who receives deferred prosecutions and complete MMT, may be withdrew and prosecuted directly by the prosecutor if any criminal case has been committed during the subsequent two-year period of probation [15].

Currently, Taiwan’s prisons have a serious over-population problem. In 2014, the estimated available space for each prisoner was only 15.3 ft^2^, far below the 24.9 ft^2^ specified by international human rights regulations [16]. Considering the addictive nature of drugs, not immediately prosecuting drug users, but first giving them an opportunity for treatment, perhaps reduce prison over-crowding and increase the space available to prisoners.

Employment status can be a critical indicator of social control, economic conditions, return to society and recidivism. Employment is an essential component of healthy social development. Critical to identity formation [17], the accumulation of social capital [18] and perceived quality of life [19], employment can enhance physical and mental well-being and overall quality of life [20, 21]. Illegal drug use is associated with poor employment outcomes. The unemployment problem of heroin users could be caused by both of the drug-abusing itself (i.e. adverse psychosocial outcomes, including disability or impairment and psychiatric comorbidity) and the social discrimination [22, 23]. For example, previous study found that the use of heroin and cocaine, as well as the higher frequency of use, is predictive of membership in a low-employment trajectory group through middle adulthood [24]. Evidence-based determinants of employment include educational attainment [25], race [26], marital status and family structure [27], socio-economic status [28] and general health status [29]. However, these studies did not explore whether the type of sentence affected subsequent employment status among illegal heroin users. Therefore, this study examines the association of sentencing types and employment status at 2 years after release among illegal heroin users in Taiwan, to determine the long-term effectiveness of alternatives to prison sentencing.

## Method

### Ethics statement

The Institutional Review Board (IRB) of National Taiwan Normal University approved this study (No. 201606HM002). Written consent from the study participants was waived because the data were collected from a population-based database of de-identified secondary data.

### Sample

This was a population-based cohort study. Illegal heroin users in the national prison register system and deferred prosecution system were identified who received a sentence from January 1, 2011 to December 31, 2011. There were 2406 deferred prosecutions with MMT requirement, 4741 observation and rehabilitation, 15 compulsory rehabilitation and 1958 sentences to prison included in this study. All study participants were followed until 2 years after the index date, that is, the date of deferred prosecution, release from prison or release from the treatment centers.

### National Health Insurance in Taiwan

In 1995, the National Health Insurance (NHI) program, a government-run, single-payer insurance system, was established in Taiwan. By December 2010, 23.074 million people were enrolled nationwide, with a coverage rate of 99.6%. The NHI Research Database (NHIRD) contains patient demographic and insurance premium data. The insurance premium was used as a proxy indicator of employment status and was classified as with or without job salary. Those with a fixed premium who were also listed as a dependent on the insurance premium were considered as not having a job salary. The fixed premium group is composed of people receiving social welfare support and includes low-income people and veterans. The dependent insurance premium group is comprised of people with family members with no job or income.

### Main outcome and study variables

Two periods were used to measure the employment status of study participants, the follow-up period and the past period. The follow-up period for monitoring employment status began at the date of deferred prosecution or release from either prison or the treatment centers and continued until 2 years after the index date. The past period for monitoring employment status was retrospective, tracking back 2 years before the index date. The past period of employment status was included in the analysis as a control variable. In our database, the minimal follow-up duration after index date for all study subjects is around 2 years. Therefore, we used 2 years as time window to analyze the employ status after index date.

For controlling potential confounders, social demography was collected from National Population Register Database, including gender, age, types of residence (rural, urban), insurance premium (with/without job salary), education level (elementary, junior high school, senior high school and college) and marital status (single, married, divorced and widowed) [22, 30, 31]. Illicit drug use criminal record collected from National Police Agency in Taiwan [32, 33]. Mental diseases including schizophrenia, depressive disorders, bipolar disorders and general health condition (Charlson comorbidity score) were collected from NHIRD [31, 34, 35]. All of the potential comorbid diseases and Charlson comorbidity score were defined using outpatient and inpatient diagnosis before the index date.

### Statistical analysis

Chi-square analysis was used to examine the differences in demographic and other characteristics of those with or without a job at 2 years after the index date. The impact of the type of sentence was analyzed using logistic regression. The un-adjusted and adjusted odds-ratio (AOR) were reported. The model was tested first using all participants, then according to employment status before sentencing. Dummy variables were created to perform collinearity diagnosis by using multiple regression, variance inflation factor (VIF) and condition index were reported. Variables with VIF > 10 or condition index> 30 was considered as collinearity problem in the study model. Analyses were carried out using SAS version 9.4 (SAS Institute Inc., Cary, NC, USA). *P* values < 0.05 were considered statistically significant.

## Results

### Sample characteristics

Results for the characteristics of illegal heroin users are summarized in Table 1. Of the 1848 participants unemployed at 2 years after the index date, 1524 were male and 324 were female. The unemployment rate was similar between males and females (*P* = 0.759) and those with rural and urban residence (*P* = 0.087), schizophrenia (*P* = 0.321), depression (*P* = 0.687), bipolar (*P* = 0.381). The unemployment rate was associated with age, prior employment status, type of sentencing, education level, marital status, illegal drug use criminal record and Charlson comorbidity score (all *P* < 0.05).

**Table 1:** Demographic and other characteristics of study participants, Taiwan, 2011 (*n* = 9120)

Characteristics	Insurance premium at two years after index date^a^		*P* value
	No job salary, *n* = 1848	With job salary, *n* = 7272	
Gender			
Male	1524 (20.20)	6019 (79.80)	0.759
Female	324 (20.55)	1253 (79.45)	
Age (years) at index date			
18–24	375 (23.32)	1233 (76.68)	< 0.001
25–44	1160 (18.44)	5131 (81.56)	
≥ 45	313 (25.63)	908 (74.37)	
Type of residence			
Rural	539 (21.43)	1976 (78.57)	0.087
Urban	1309 (19.82)	5296 (80.18)	
Insurance premium at two years after index date			
With job salary	1265 (16.01)	6635 (83.99)	< 0.001
Without job salary	583 (47.79)	637 (52.21)	
Type of sentencing			
Deferred prosecution	393 (16.33)	2013 (83.67)	< 0.001
Observation and rehabilitation	853 (17.99)	3888 (82.01)	
Compulsory rehabilitation	6 (40.00)	9 (60.00)	
Sentenced to prison	596 (30.44)	1362 (69.56)	
Education level			
Elementary	323 (21.72)	1164 (78.28)	< 0.001
Junior high school	1107 (21.47)	4050 (78.53)	
Senior high school	383 (16.89)	1885 (83.11)	
College	35 (16.83)	173 (83.17)	
Marital status			
Single	1335 (19.30)	5581 (80.70)	< 0.001
Married	348 (21.97)	1236 (78.03)	
Divorce or widowed	165 (26.61)	455 (73.39)	
Drug use criminal record			
0	236 (18.02)	1074 (81.98)	< 0.001
1	559 (15.23)	3111 (84.77)	
2	342 (19.94)	1373 (80.06)	
≥ 3	711 (29.32)	1714 (70.68)	
Schizophrenia			
Yes	1822 (20.31)	7147 (79.69)	0.321
No	26 (17.22)	125 (82.78)	
Depression			
Yes	1675 (20.20)	6616 (79.80)	0.687
No	173 (20.87)	656 (79.13)	
Bipolar			
Yes	1819 (20.22)	7179 (79.78)	0.381
No	29 (23.77)	93 (76.23)	
Charlson comorbidity score			
0	915 (21.43)	3354 (78.57)	< 0.001
1	446 (18.82)	1924 (81.18)	
2	206 (16.78)	1022 (83.22)	
≥ 3	281 (22.43)	972 (77.57)	

^a^ The index date was the date of release from prison, treatment centers or deferred prosecution; job status monitoring lasted for two years after the index date

### Risk factors associated with unemployment status among illegal heroin users

After controlling for gender, age, type of residence, previous employment status, education level, marital status, illegal drug use criminal record, schizophrenia, depression, bipolar and Charlson comorbidity score, the unemployment rate at 2 years of those sentenced to prison was 2.30 times that of those who received deferred prosecution (AOR = 2.30, 95% Confidence Interval [CI]: 1.97–2.65, *P* < 0.001). The unemployment rate was also higher for those with observation and rehabilitation and compulsory rehabilitation than deferred prosecution in the 2 years following sentencing. Males, older users, without a job before sentencing, divorced or widowed and higher prior illegal drug use criminal records were also higher risk of unemployment. With senior high school educational level was lower significant risk of unemployment. The strength of associations showed dose-dependent relationship between different sentencing types (sentenced to prison> compulsory rehabilitation> observation and rehabilitation) and employment outcomes (all *P* < 0.001, Table 2). In this study, the dysfunction of mental (schizophrenia, depression and bipolar) and general health condition (Charlson comorbidity score) were not significantly associated with unemployment rate in illegal heroin users (all *p* > 0.05, Table 2).

**Table 2:** Logistic regression analysis of odds of unemployment at two years after release following heroin use sentence in Taiwan, 2011 (*n* = 9120)

Variable	Un-adjusted odds-ratio		Adjusted odds-ratio^b^		VIF^c^
	Estimate (95% CI)	*P* value	Estimate (95% CI)	*P* value	
**Social demography**^**d**^					
Gender					
Male	1.00		1.00		
Female	1.02 (0.89–1.17)	0.758	0.75 (0.64–0.87)	< 0.001	1.1
Age (years) at index date^a^					
18–24	1.00		1.00		
25–44	0.74 (0.65–0.85)	< 0.001	1.41 (1.17–1.70)	< 0.001	2.2
≥ 45	1.13 (0.95–1.35)	0.155	1.55 (1.21–1.98)	< 0.001	2.3
Type of residence					
Rural	1.00		1.00		
Urban	0.91 (0.81–1.01)	0.087	0.91 (0.80–1.03)	0.131	1.0
Insurance premium at 2 years before index date					
With job salary	1.00		1.00		
Without job salary	4.8 (4.23–5.45)	< 0.001	6.65 (5.67–7.82)	< 0.001	1.2
Education level					
Elementary	1.00				
Junior high school	0.99 (0.85–1.14)	0.844	1.06 (0.91–1.25)	0.448	2.0
Senior high school	0.73 (0.62–0.87)	< 0.001	0.82 (0.68–1.00)	0.049	2.2
College	0.73 (0.49–1.09)	0.122	0.82 (0.54–1.25)	0.358	1.1
Marital status					
Single	1.00		1.00		
Married	1.18 (1.03–1.35)	0.019	1.06 (0.9–1.24)	0.500	1.2
Divorced or widowed	1.52 (1.25–1.84)	< 0.001	1.42 (1.13–1.78)	0.003	1.2
Drug use sentences and criminal record^d^					
Type of sentencing					
Deferred prosecution	1.00		1.00		
Observation and rehabilitation	1.12 (0.99–1.28)	0.081	1.31 (1.13–1.52)	< 0.001	1.6
Compulsory rehabilitation	2.24 (1.94–2.59)	< 0.001	1.87 (1.59–2.20)	< 0.001	1.5
Sentenced to prison	3.41 (1.21–9.65)	0.021	2.30 (1.97–2.65)	< 0.001	1.0
Drug use criminal record					
0	1.00		1.00		
1	0.86 (0.69–1.07)	0.121	0.88 (0.73–1.06)	0.171	2.3
2	1.13 (0.94–1.37)	0.205	1.15 (0.93–1.4)	0.191	1.9
≥ 3	1.88 (1.59–2.23)	< 0.001	1.90 (1.56–2.32)	< 0.001	2.5
Mental diseases and comorbidity^d^					
Schizophrenia (Yes vs No)	0.80 (0.51–1.24)	0.322	0.86 (0.54–1.39)	0.542	1.1
Depression (Yes vs No)	1.04 (0.87–1.25)	0.684	1.08 (0.87–1.34)	0.509	1.3
Bipolar (Yes vs No)	1.21 (0.79–1.87)	0.381	1.33 (0.82–2.16)	0.242	1.1
Charlson comorbidity score					
0	1.00		1.00		
1	0.94 (0.85–1.07)	0.115	0.90 (0.79–1.04)	0.153	1.2
2	0.94 (0.82–1.08)	0.111	0.80 (0.66–0.96)	0.017	1.2
≥ 3	1.06 (0.91–1.24)	0.460	0.99 (0.82–1.18)	0.888	1.3

^a^ The index date was the date of release from prison, treatment centers or deferred prosecution

^b^ Global model fit Wald test x^2^ = 807.6, DF = 22, *P* < 0.001; R-square = 0.154

^c^ *VIF* Variance inflation factor

^d^For controlling potential confounders, social demography were collected from National Population Register Database, including gender, age, types of residence, insurance premium, education level and marital status [22, 30, 31] . Illicit drug use criminal record collected from National Police Agency in Taiwan [32, 33]. Mental diseases including schizophrenia, depressive disorders, bipolar disorders and general health condition (Charlson comorbidity score) were collected from NHIRD [31, 34, 35]

### Stratified analysis by prior unemployment status

There was a significant interaction between prior employment status and type of sentence (*P* < 0.001). To determine the relationship between type of sentence and the prior employment status of participants, we performed stratification analysis. For participants without a job before the index date, the unemployment rate at 2 years after the index date was not significant difference in type of sentencing (Table 3). However, at 2 years after sentencing, for those with a job before the index date, the unemployment rate for those who received a prison sentence was higher than for those who received deferred prosecution (AOR = 4.63, 95% CI: 1.22–17.57, *P* = 0.024). In addition, males, participants with divorced or widowed and ≥ 3 illegal drug use criminal record were also higher risk of unemployment (*P* < 0.001), but age and type of residence were not associated with subsequent employment status (Table 4).

**Table 3:** Logistic regression analysis of odds of unemployment at two years after release following heroin use sentence in Taiwan for those unemployed before sentencing, 2011 (*n* = 1220)

Variable	Un-adjusted odds-ratio		Adjusted odds-ratio^b^		VIF^c^
	Estimate (95%CI)	*P* value	Estimate (95%CI)	*P* value	
**Social demography**^**d**^					
Gender					
Male	1.00		1.00		
Female	0.94 (0.74–1.19)	0.610	0.97 (0.74–1.26)	0.815	1.1
Age (years) at index date^a^					
18–24	1.00		1.00		
25–44	1.79 (1.40–2.3)	< 0.001	1.98 (1.40–2.80)	< 0.001	1.9
≥ 45	4.34 (2.9–6.49)	< 0.001	5.10 (2.79–9.35)	< 0.001	2.3
Type of residence					
Rural	1.00		1.00		
Urban	0.88 (0.68–1.15)	0.345	0.87 (0.65–1.15)	0.331	1.0
Education level					
Elementary	1.00		1.00		
Junior high school	0.87 (0.66–1.14)	0.311	0.87 (0.65–1.16)	0.348	1.4
Senior high school	1.37 (0.92–2.05)	0.118	0.91 (0.57–1.46)	0.706	1.7
College	1.03 (0.40–2.66)	0.956	0.59 (0.21–1.64)	0.312	1.1
Marital status					
Single	1.00		1.00		
Married	1.89 (1.38–2.59)	< 0.001	0.87 (0.57–1.33)	0.529	1.7
Divorced or widowed	2.43 (1.38–4.29)	0.002	0.95 (0.47–1.90)	0.888	1.5
Drug use Sentences and criminal record^d^					
Type of sentencing					
Deferred prosecution	1.00		1.00		
Observation and rehabilitation	0.89 (0.67–1.18)	0.414	1.19 (0.86–1.63)	0.287	1.6
Compulsory rehabilitation	0.71 (0.12–4.33)	0.711	0.90 (0.13–6.15)	0.914	1.6
Sentenced to prison	1.34 (0.93–1.94)	0.118	1.04 (0.68–1.60)	0.849	1.0
Drug use criminal record					
0	1.00		1.00		
1	0.90 (0.65–1.25)	0.534	0.89 (0.63–1.26)	0.513	2.0
2	1.11 (0.76–1.61)	0.595	1.00 (0.67–1.49)	0.984	1.8
≥ 3	1.05 (0.73–1.53)	0.787	0.86 (0.57–1.29)	0.464	2.0
Mental diseases and comorbidity^d^					
Schizophrenia (Yes VS No)	3.22 (0.33–6.00)	0.312	1.17 (0.11–2.09)	0.894	1.1
Depression (Yes VS No)	2.41 (1.51–3.84)	< 0.001	2.44 (1.42–4.19)	0.001	1.3
Bipolar (Yes VS No)	1.94 (0.65–5.84)	0.236	0.79 (0.22–2.80)	0.720	1.2
Charlson comorbidity score					
0	1.00		1.00		
1	1.15 (0.87–1.52)	0.336	1.11 (0.83–1.49)	0.480	1.1
2	1.03 (0.70–1.52)	0.871	0.90 (0.60–1.36)	0.626	1.1
≥ 3	1.87 (1.26–2.79)	0.002	1.14 (0.71–1.84)	0.590	1.4

^a^ The index date was the date of release from prison, treatment centers or deferred prosecution

^b^ Global model fit Wald test x^2^ = 72.28, DF = 21, *P* < 0.001; R-square = 0.093

^c^ *VIF* Variance inflation factor

^d^For controlling potential confounders, social demography were collected from National Population Register Database, including gender, age, types of residence, insurance premium, education level and marital status [22, 30, 31]. Illicit drug use criminal record collected from National Police Agency in Taiwan [32, 33]. Mental diseases including schizophrenia, depressive disorders, bipolar disorders and general health condition (Charlson comorbidity score) were collected from NHIRD [31, 34, 35]

**Table 4:** Logistic regression analysis of risk of unemployment at two years after release following heroin use sentence in Taiwan among those employed before sentence, 2011 (*n* = 7900)

Variable	Un-adjusted odds-ratio		Adjusted odds-ratio^b^		VIF ^c^
	Estimate (95%CI)	*P* value	Estimate (95%CI)	*P* value	
**Social demography**^**d**^					
Gender					
Male	1.00		1.00		
Female	0.61 (0.5–0.74)	< 0.001	0.63 (0.51–0.77)	< 0.001	1.1
Age (year) at index date^a^					
18–24	1.00		1.00		
25–44	1.36 (1.1–1.67)	0.004	1.11 (0.88–1.40)	0.364	2.2
≥ 45	1.7 (1.33–2.18)	< 0.001	1.05 (0.78–1.41)	0.752	2.5
Type of residence					
Rural	1.00		1.00		
Urban	0.85 (0.75–0.97)	0.018	0.91 (0.77–1.12)	0.208	1.0
Education level					
Elementary	1.00		1.00		
Junior high school	1.29 (1.07–1.56)	0.007	1.22 (1–1.48)	0.051	2.2
Senior high school	0.98 (0.80–1.22)	0.888	0.90 (0.71–1.12)	0.339	2.3
College	0.95 (0.60–1.51)	0.825	0.94 (0.58–1.51)	0.791	1.2
Marital status					
Single	1.00		1.00		
Married	1.07 (0.90–1.26)	0.452	1.06 (0.89–1.27)	0.517	1.2
Divorced or widowed	1.60 (1.29–1.98)	< 0.001	1.51 (1.19–1.94)	0.001	1.2
Drug use Sentences and criminal record^d^					
Type of sentencing					
Deferred prosecution	1.00		1.00		
Observation and rehabilitation	1.02 (0.87–1.19)	0.841	1.38 (1.16–1.65)	< 0.001	1.5
Compulsory rehabilitation	2.61 (2.22–3.08)	< 0.001	2.07 (1.73–2.47)	< 0.001	1.5
Sentenced to prison	4.62 (1.29–16.47)	0.018	4.63 (1.22–17.57)	0.024	1.0
Drug use criminal record					
0	1.00		1.00		
1	0.87 (0.70–1.08)	0.211	0.87 (0.70–1.09)	0.237	2.3
2	1.25 (0.98–1.59)	0.070	1.19 (0.93–1.52)	0.161	1.9
≥ 3	2.67 (2.16–3.03)	< 0.001	2.30 (1.82–2.90)	< 0.001	2.6
Mental diseases and comorbidity^d^					
Schizophrenia (Yes VS No)	0.95 (0.59–1.51)	0.816	0.92 (0.56–1.52)	0.750	1.1
Depression (Yes VS No)	0.91 (0.73–1.14)	0.413	0.88 (0.68–1.13)	0.313	1.3
Bipolar (Yes VS No)	1.15 (0.69–1.91)	0.605	1.38 (0.79–2.41)	0.254	1.1
Charlson comorbidity score					
0	1.00		1.00		
1	0.81 (0.69–0.95)	0.008	0.84 (0.71–0.98)	0.031	1.2
2	0.73 (0.59–0.89)	0.002	0.77 (0.62–0.94)	0.013	1.2
≥ 3	1.06 (0.88–1.27)	0.529	0.94 (0.77–1.15)	0.569	1.3

^a^ The index date was the date of release from prison, treatment centers or deferred prosecution

^b^ Global model fit Wald test x^2^ = 368.5, DF = 21, *P* < 0.001; R-square = 0.085

^c^ *VIF* Variance inflation factor

^d^For controlling potential confounders, social demography were collected from National Population Register Database, including gender, age, types of residence, insurance premium, education level and marital status [22, 30, 31]. Illicit drug use criminal record collected from National Police Agency in Taiwan [32, 33]. Mental diseases including schizophrenia, depressive disorders, bipolar disorders and general health condition (Charlson comorbidity score) were collected from NHIRD [31, 34, 35]

### Collinearity diagnosis

All the VIFs for models less than 10 (Tables 2, 3 and 4) and condition index less than 30 (Table 5) in this study, the results showed mild collinearity problem in our study models.

**Table 5:** Condition index to check collinearity for the models of Tables 2, 3 and 4 in this study

Number	Condition Index		
	Model of Table 2	Model of Table 3	Model of Table 4
1	1.0	1.0	1.0
2	2.0	1.9	2.0
3	2.3	2.1	2.3
4	2.5	2.2	2.5
5	2.6	2.4	2.7
6	2.7	2.5	2.7
7	2.7	2.6	2.7
8	2.7	2.6	2.7
9	2.8	2.6	2.8
10	2.8	2.7	2.8
11	2.8	2.8	2.8
12	2.9	2.9	2.9
13	3.0	3.0	3.0
14	3.0	3.2	3.1
15	3.1	3.4	3.7
16	3.7	3.6	3.8
17	3.8	3.8	3.9
18	3.9	4.1	4.4
19	4.4	5.4	5.3
20	5.4	7.3	8.9
21	8.4	9.3	10.5
22	10.3	19.4	21.8
23	21.4	–	–

## Discussion

In the present study, after controlling potential confounders (age, insurance premium at 2 years after index date, education level, marital status, illegal drug use criminal record, Charlson comorbidity score), the unemployment rate of illegal heroin users sentenced to prison was higher than that for those who received deferred prosecution, observation and rehabilitation and compulsory rehabilitation. These results indicate that, in the 2 years after release from the penal system, sentencing to prison made it more difficult for illegal heroin users to acquire a job compared to counterparts of the other three types. Employment is a critical component of the reentry process that positively assists illegal drug users to return to the community. The results support the viewpoint that a deferred prosecution policy can decrease the risk of unemployment and subsequently improve offenders’ social control, economic conditions and ability to return to society. Continued implementation of a deferred prosecution and complete MMT treatment therefore benefits the offender and solve the problem of prison overcrowding.

Employment is a fundamental element of social control, providing a daily routine and expectations regarding performance and productivity. Whether illegal substance use disrupts that process and is associated with negative outcomes is therefore a critical social, economic, and criminal justice question, with implications for drug policy. Although the importance of the issue is widely recognized, the strength and direction of the relationship remains unclear. Early research often reported that drug use is unrelated to employment outcomes [36, 37] and, in some instances, that drug use is associated with a higher wages [38]. In contrast, research conducted post-2000 reveals, more often than not, that drug use is associated with poor employment outcomes. Huang et al. [24] find that use of heroin as well as higher frequency use is predictive of membership in a low-employment trajectory group through middle adulthood. The present study extended these findings and further showed that higher illegal drug use criminal record (> 3 times) was associated with a risk of unemployment after controlling potential confounders, especially those who had a job before sentencing.

As well, DeSimone [39], adopting a sophisticated econometric model that addresses simultaneity, reports a negative relationship between illegal drug use, employment, and labor force participation [40]. Problematic substance use increases the likelihood of unemployment and decreases the chance of finding and holding down a job. Unemployment is also a significant risk factor for substance use and the subsequent development of substance use disorders. Unemployment and substance use have a bidirectional correlation [41]; in other words, improving the employment rate in the illegal drug use population contribute to reduce the recurrence of drug use. Moreover, employment is highly associated with treatment retention [42, 43]. Thus, the deferred sentencing model can decrease the unemployment rate and assist former drug users continue to receive treatment. Employment helps users maintain their treatment and recover better.

A previous study revealed that former employment and work experiences are protective factors that increase the likelihood of employment after release [44]. Similarly, our data showed that the unemployment rate for illegal heroin users without a job before the index date was higher than that for those with a job before the index data. Interestingly, those sentenced to prison had a higher risk of unemployment than those who received deferred prosecution, observation and rehabilitation and compulsory rehabilitation, even if they were employed before being arrested (Tables 3 and 4). This result suggests that illegal heroin use treatment for those with prior employment might be superior in the criminal justice settings to sentencing only, but not for those previously were unemployed. Reducing the number of people incarcerated for illegal drug use can net huge savings in economic and social costs [45]. The finding support that the policy of prioritizing treatment for illegal heroin user over imprisonment deserves continued promotion.

Previous studies have indicated that gender and age are associated with employment status in illegal heroin user [24, 46]. At ages 23–25, the low-employment trajectory group was employed in approximately 21 of 52 workweeks, followed by a rapid decline to 10 or fewer by age 40 [24]. Similarly, we also found that being male and being older increased the risk of unemployment in illegal heroin users at 2 years after release. Furthermore, we found that the association of gender and employment status remained significant for those previously were employed, while the association of age and employment status was still significant for those previously were unemployed. This difference suggests that previously unemployed older heroin users and previously employed male users were more likely to be jobless after arrest, increasing their likelihood of unemployment after their release from the system.

A illegal drug use crime refers to a detrimental behavior or an illness, society has a obligation to expect that an effective public policy or approach to the “illegal drug use problem” that will reduce drug-related crime, unemployment, family dysfunction and disproportionate use of heath care [47]. However, although half of prisoners need treatment, approximately 14.8–17.4% reported having received drug treatment since admission in a survey of US State and Federal prisoners, according to the Bureau of Justice Statistics (BJS) [48]. Another study was conducted to compare rates of employment before, during, and after employment at the therapeutic workplace [49]. The researchers found that unemployed chronic drug users will attend work at higher rates at the therapeutic workplace than in the community when paid modest wages. Thus, after receiving treatment, a successful mechanism that can transfer the illegal drug user to the therapeutic workplace contribute to improve their employment rate. Providing vocational training as part of drug abstinence-oriented treatment has the potential to improve employment-seeking behaviors, increase the chances of securing employment, improve the employment rate and lead to higher paying jobs [48]. Consequently, a high quality treatment program is necessary and should produce positive results.

To our knowledge, this is the first national population-based study to investigate the association of type of sentencing and the subsequent employment status among illegal heroin users in Taiwan. The major strength of this study is its population-based study design. Because this study was conducted using the population-based database, the findings can be applied to the general population. However, this study has several limitations. Several crucial confounding factors could not be included in this study, including severity of the substance use disorder, family income and social support system since our study database did not contain this information. Second, we only track the employment status of illegal heroin users at 2 years after release, if more time points of employment status can be included in the analyses, generalizability of study findings will be warrant. Third, for drug users, three factors associated with unemployed were lower education, the personal willing of discouraged workers, and social discrimination. As Webster et al. (2007) addressed that all of the employers surveyed were willing to hire someone having no criminal record before hiring a released prisoner [33]. However, there is no relevant survey variable in the database, the influences of these three factors did not include in this analyses. Interpretation of the study results should be with caution.

## Conclusion

Basically, illegal heroin users are legally regarded as a patient before being regarded as a criminal, so giving priority to quit addition rather than imprisonment. This study supports this viewpoint that staying in the community to receive methadone maintenance treatment (MMT) may increase the odds ratio of illegal heroin users who keep their jobs. But for illegal heroin users who were unemployed before their arrest would not be significantly different because of four different sentencing types. Illegal heroin users sent to prison may be at greater risk for subsequent unemployment than those receiving deferred prosecution, observation and rehabilitation, and compulsory rehabilitation, especially among those who had a job before sentencing. The implication is the stronger freedom of punishment, the higher risk of unemployment outcomes. In addition, males, older offenders, those without a job before sentencing, divorced or widowed, those have ≥3 illegal drug use criminal records are also at higher risk of unemployment at 2 years after release. Effective public policy should strive to increase the employability of this population, in order to reduce both illegal drug use and prison sentencing in society as a whole. However, such a conclusion of the causal relationship between the deferred prosecution policy and the risk of unemployment should be proved by the social experiments or other methods for further research.

## Data Availability

The data used to support the findings of this study are included within the article.

## Abbreviations

BJS: Bureau of Justice Statistics
HIV: Human immunodeficiency virus
IRB: Institutional Review Board
MMT: Methadone maintenance treatment
NHI: National Health Insurance
NHIRD: NHI Research Database
